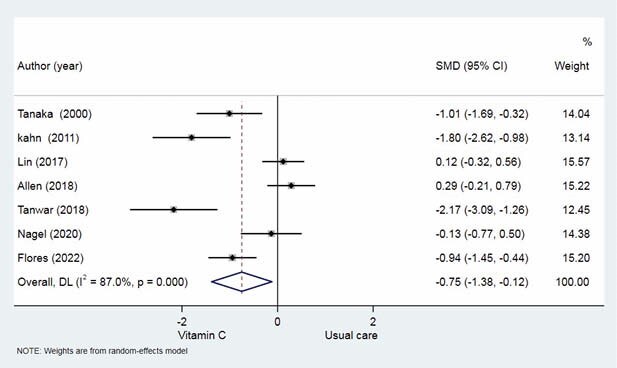# 57 Effect of High-Dose Ascorbic Acid in Major Burn Patients: A Systematic Review and Meta-Analysis

**DOI:** 10.1093/jbcr/irad045.031

**Published:** 2023-05-15

**Authors:** Punthiti Pleehachinda, Pornprom Muangman, Kusuma Chinaroonchai

**Affiliations:** Faculty of Medicine Siriraj Hospital, Mahidol University, Bangkok, Krung Thep; Faculty of Medicine Siriraj Hospital, Mahidol University, Bangkok, Krung Thep; Faculty of Medicine Siriraj Hospital, Mahidol University, Bangkok, Krung Thep

## Abstract

**Introduction:**

High-dose ascorbic acid (HDAA) plays an important role in oxygen radical elimination which is a major cause of pathophysiological derangement in burn patients. However, there was limited explicit data on the clinical effect of HDAA in major burn patients. The purpose of this study was to identify the benefit and risks of HDAA in major burn patients.

**Methods:**

MEDLINE, EMBASE, and Cochrane database searches were conducted. An additional search of relevant primary literature and review articles was performed. All the studies related to HDAA and burn were included in the analysis. HDAA group was defined by receiving ascorbic acid 66 mg/kg/day in the first 24 hours. The primary outcome was the 24-hour fluid requirement. The secondary outcomes were in-hospital mortality, 24-hour urine output, ventilator day, hospital length of stay, and in-hospital complications. Pooled incidences and 95% confidence interval (CI) were calculated and summarized using a random effect model on Stata/MP 16.1.

**Results:**

Of 875 studies, 7 articles (293 patients) were included in this meta-analysis. HDAA significantly reduced 24-hour fluid requirement compared with usual care (no HDAA) (SMD -0.75 ml/kg per TBSA, 95% CI [-1.38 to -0.12]). HDAA group showed a decrease in 24-hour urine output (SMD, 0.65; 95% CI, 0.41 to 0.89). However, it did not demonstrate significant increase in acute kidney injury (RR 1.37, 95% CI [0.60 to 3.13]) and the need for renal replacement therapy (RR 1.31, 95% CI [0.50 to 3.44]). In terms of in-hospital complications, there was no significant difference in abdominal compartment syndrome (RR 0.22, 95% CI [0.08 to 0.89]) and pneumonia or acute respiratory distress syndrome (RR 1.03, 95% CI [0.70 to 1.51]). Though there was a trend to decrease mortality in the HDAA group, there was no statistical difference in mortality (RR 1.27, 95% CI [0.85 to 1.91]). HDAA group had a comparable hospital length of stay (SMD 0.1, 95% CI [-0.44 to 0.63]) and ventilator day (SMD 0.03, 95% CI [-0.64 to 0.70]).

**Conclusions:**

High-dose ascorbic acid had a clinical benefit in the significant reduction of 24-hour fluid requirement without increasing the risk for acute kidney injury or the need for renal replacement therapy. Though it did not demonstrate a significant improvement in mortality or length of stay, there were no significant reported complications from high-dose ascorbic acid. A multicenter randomized controlled trial is warranted.

**Applicability of Research to Practice:**

The potential benefit of HDAA in major burn